# The Glycolytic Pathway as a Target for Novel Onco-Immunology Therapies in Pancreatic Cancer

**DOI:** 10.3390/molecules26061642

**Published:** 2021-03-15

**Authors:** Claudia Curcio, Silvia Brugiapaglia, Sara Bulfamante, Laura Follia, Paola Cappello, Francesco Novelli

**Affiliations:** 1 Department of Molecular Biotechnology and Health Sciences, University of Turin, 10126 Turin, Italy; claudia.curcio@unito.it (C.C.); silvia.brugiapaglia@unito.it (S.B.); sara.bulfamante@unito.it (S.B.); laura.follia@unito.it (L.F.); paola.cappello@unito.it (P.C.); 2 Centro Ricerche Medicina Sperimentale, Azienda Ospedaliera Universitaria, Città della Salute e della Scienza di Torino, 10126 Turin, Italy; 3 Computer Science Department, University of Turin, 10126 Turin, Italy

**Keywords:** pancreatic cancer, glycolysis, immunotherapies

## Abstract

Pancreatic ductal adenocarcinoma (PDA) is one of the most lethal forms of human cancer, characterized by unrestrained progression, invasiveness and treatment resistance. To date, there are limited curative options, with surgical resection as the only effective strategy, hence the urgent need to discover novel therapies. A platform of onco-immunology targets is represented by molecules that play a role in the reprogrammed cellular metabolism as one hallmark of cancer. Due to the hypoxic tumor microenvironment (TME), PDA cells display an altered glucose metabolism—resulting in its increased uptake—and a higher glycolytic rate, which leads to lactate accumulation and them acting as fuel for cancer cells. The consequent acidification of the TME results in immunosuppression, which impairs the antitumor immunity. This review analyzes the genetic background and the emerging glycolytic enzymes that are involved in tumor progression, development and metastasis, and how this represents feasible therapeutic targets to counteract PDA. In particular, as the overexpressed or mutated glycolytic enzymes stimulate both humoral and cellular immune responses, we will discuss their possible exploitation as immunological targets in anti-PDA therapeutic strategies.

## 1. Introduction

Pancreatic ductal adenocarcinoma (PDA) is one of the most lethal forms of cancer and is characterized by rapid progression, invasiveness and treatment resistance. In future decades, it is predicted to become the second leading cause of cancer death with an overall survival of ~10% [1]. At the time of diagnosis, only 20% of patients were suitable for surgery which is, currently, is the only potentially curative treatment [2].

**Figure 1:**
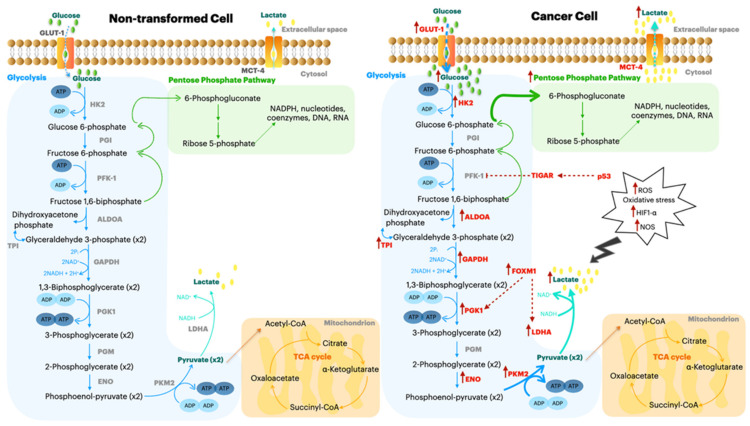
Schematic comparison of metabolic pathways between a nontransformed cell and a cancer cell. The enzymes, transcription factors, oncogenes involved in aberrant glycolysis and correlated pathways are highlighted in red and are discussed in detail in the text.

One of the main characteristics that causes aggressiveness in PDA is aberrant glucose metabolism. Indeed, in PDA cells, most of the glucose molecules are quickly transformed into lactate, and glucose becomes the main energy source for the tumor. This mechanism is known as the Warburg effect or aerobic glycolysis [3], whereby cancer cells favor metabolism via glycolysis to satisfy the increasing needs of nucleotide, lipid and protein synthesis required for cell replication (Figure 1).

The enhanced glycolysis and the consequent decrease in oxidative phosphorylation lead to higher lactate production, triggering an acidic and immunosuppressive tumor microenvironment (TME) [4,5,6,7,8]. In this context, the glycolysis rate of T cells is reduced in the tumor niche resulting in inhibition of T cell proliferation and cytokine production [7], favoring tumor immune evasion [8]. Furthermore, the immune response against some glycolytic enzymes was detected in PDA patients [9,10].

In this review, we provide a description of the main glycolytic enzymes that are overexpressed in PDA, highlighting when an immune response was demonstrated, in order to propose suitable targets to delay tumor growth (Table 1). 

## 2. KRAS Aberrant Signaling and TP53 Mutation in PDA

KRAS mutations are the first events found in most PDA patients [11,12,105]. Constitutive KRAS activation drives high proliferation of PDA cells, which need the right amount of energy and nutrients to quickly replicate. Many studies have demonstrated the active role of RAS oncogenes in fueling and upregulating glycolysis [11,20,106]. One of the first effects of KRAS activation is enhanced transcription of glucose transporter 1 (GLUT1), leading to both an increased glycolytic activity and lactate production. Together with GLUT1, mutant KRAS caused an increase in expression of several glycolytic enzymes such as hexokinase 2 (HK2), phosphofructokinase 1 (PFK1) and lactate dehydrogenase A (LDHA) [20]. Aberrant KRAS signaling shunted glycolytic intermediates into the hexosamine biosynthetic pathway (HBP) and pentose phosphate pathway (PPP), which provided precursors for post-translational modifications of proteins and biosynthesis of nucleic acids [105].

Several therapeutical approaches have been developed to target KRAS activation in different malignancies. KRAS inhibitors have shown promising results in nonsmall cell lung cancer patients, while only a few colorectal cancer patients were responsive to therapy, suggesting tissue-specific applicability of KRAS inhibition [107]. In PDA, an interesting approach to neutralize mutated KRAS was achieved by using a monoclonal antibody (mAb), RT11-i, for targeting the intracellular activated GTP-bound form of oncogenic RAS mutants [108]. In vitro and in preclinical models, RT11-i enhanced the antitumor activity of gemcitabine (GEM), the most commonly used chemotherapeutic drug for PDA, suggesting that the use of combined treatment is a potential strategy for patients with KRAS mutations [108]. Unfortunately, 30% of PDA cases were able to escape KRAS inhibition [11], thus targeting KRAS with peptides containing different mutations could be an important strategy. In fact, different KRAS mutations were found in gastrointestinal malignancies, thus peptide vaccination targeting these mutations has been used both in mouse models and in clinical trials. This vaccination has not yet provided clinical benefits [11,14,109], but clinical trials with new peptides are still in progress [110,111] (NCT04117087, NCT00005630, phase 1). Conversely, peptide vaccination in a colon cancer mouse model did elicit antitumor effects mediated by the T helper 1 (Th1) immune response, an increased percentage of effector CD8^+^ T cells and a decrease in immune suppressive T cells [19]. Moreover, one of the most important effects of KRAS activation in PDA cells is the alteration of the TME—for example, IL-6 and TGF-β secretion leading to the inflammatory response, tumor growth and regulation of tumor stroma [11]. Additional studies are required to better clarify the effect of KRAS activation on immune cells.

A consequence of KRAS activation is the genetic inactivation of the TP53 tumor suppressor pathway [11]. Inactivation of TP53 supported glycolysis by impairing the nuclear translocation of the glycolytic enzyme glyceraldehyde-3-phosphate dehydrogenase (GAPDH), whose levels increased in the cytosol [112], increasing tumor aggressiveness with a higher activity of anabolic pathways [113]. These pathways are supported by autophagy; therefore, treatment with hydroxychloroquine, a known autophagy inhibitor, increased PDA cell proliferation in mice with KRAS mutation and loss of TP53 [113]. Preclinical models have shown that genetic ablation of autophagy increased tumorigenesis, while impairing cancer progression and invasiveness, demonstrating that autophagy could be clinically relevant in PDA treatment [114].

As for KRAS, vaccination against peptides derived from mutant p53 are being studied [110]. Currently, a combination of long peptides derived from mutated p53 and KRAS is effective to elicit T cells to target tumors [110]. Since antibodies against KRAS or p53 were found in patients with gastrointestinal, colon and lung malignancies [115,116,117], it could also be interesting to investigate the antibody response in PDA patients.

In a recent ongoing clinical trial (NCT03524677), the relationship between the four major genes—KRAS, CDKN2A, SMAD4, TP53—involved in PDA progression and preoperative staging was investigated in order to better stratify patients for setting an optimal therapeutic strategy.

### TP53 Induced Glycolysis Regulatory Phosphatase (TIGAR)

A target protein of p53 is TIGAR, a glycolytic enzyme that plays a controversial role in cancer metabolism [118]. TIGAR is a bisphosphatase activity protein that inhibits glycolysis by shifting metabolic intermediates to the alternative PPP in response to oxidative stress. This is an important step in maintaining redox balance by reducing intracellular reactive oxygen species (ROS) with an enhanced production of NADPH [119].

High expression of TIGAR has been found in many cancers and also in the TP53 wild type PDA patient-derived xenograft (PDX), while its expression is low in TP53 mutant PDX [118]. The critical role of TIGAR in the dynamic control of ROS during PDA development has been investigated in a mouse model [120]. ROS accumulation due to loss of TIGAR led to fewer premalignant pancreatic intraepithelial neoplasia (PanIN) lesions during the earliest phases of tumorigenesis, but at the same time ROS accumulation increases the ability to metastasize and invade, especially to the lungs, through the acquisition of a more mesenchymal phenotype and the activation of ERK signaling [120]. In fact, high levels of TIGAR during the initial phases of PDA and the decrease during the invasive phase suggest the presence of a temporal control of ROS accumulation resulting in a switch from a proliferative to an invasive phenotype [120]. These data are useful for optimizing the therapies targeting ROS, also considering tumor development.

## 3. Glucose Transporter 1 (GLUT1)

The increase in glucose uptake as an important hallmark in cancers has led to a focus on GLUT1, a membrane protein, predominantly expressed in the brain, erythrocytes, muscles, liver and adipocytes [121], which facilitate basal glucose uptake in most cell types. GLUT1 expression is increased in many types of cancers, including PDA [15,16,17,18,122,123,124,125], and it positively correlates with tumor grade [18]. GLUT1 functions as a passive energy-independent carrier that transports glucose through a concentration gradient, so an increased expression leads to an increased entry of glucose, accelerating cancer cell proliferation and metastasis [17,21,126]. In human tissues, GLUT1 expression increased from low- to high-grade dysplastic lesions and caused larger tumors, suggesting that GLUT1 overexpression is associated with PDA invasiveness [17], poor prognosis, lower survival and worse response to neoadjuvant chemoradiotherapy [22].

The prognostic value of GLUT1 is that it provides a noninvasive diagnostic tool based on glucose metabolism [127]. As glucose transporters (including GLUT1) are responsible for 18F-fluorodeoxyglucose (FDG) accumulation in PDA basal microvilli, structures that are not present in the microvasculature of normal tissues [128], the use of FDG positron emission tomography (FDG-PET) could predict outcomes and early recurrence after surgery in PDA patients [129]. A phase 1 clinical trial (NCT01050283) aims to evaluate if FDG-PET, in association with GEM-based therapy, can be used to screen for the activity of novel PDA treatments. Another ongoing phase 1 clinical trial (NCT04542291) evaluates the feasibility and safety of sodium glucose transporter 2 inhibitor in addition to chemotherapy for treating metastatic PDA patients.

It is well known that inducing and maintaining the antitumor immune response (both antibody and cellular) may be relevant to setting up an effective immunotherapeutic strategy. Regarding the antibody response, overexpression of certain glycolytic enzymes discussed here was associated with the presence of specific autoantibodies in cancer patients’ sera, and their detection in PDA should be investigated. The presence of autoantibodies could be diagnostic but also remission/progression markers. To date, despite its upregulation, anti-GLUT1 antibodies in the sera of cancer patients were not detected. Among immune cells, a higher expression of GLUT1 was detected in activated T regulatory cells (Tregs) in the TME [27]. In Tregs, a substantial proportion of glucose is converted into pyruvate for mitochondrial oxidization [130], and glucose depletion is unfavorable for Treg proliferation [27]. GLUT1 expression was also found to be correlated with the density of PD1^+^ tumor infiltrating lymphocytes (TILs) in specimens from PDA patients [18]. This increase is a glycolysis-dependent mechanism, since silencing of PFK1, a rate-limiting glycolytic enzyme, did not affect tumor growth in mice, but significantly reduced PD1^+^ TILs [18].

All these considerations highlight that GLUT1 is involved in PDA development and thus upstream pathways may be potential therapeutic targets. Notably, silencing of TWIST1, a highly conserved transcription factor upregulated in PDA tissues and associated with reduced overall survival in patients, reduces glucose uptake, lactate production and the extracellular acidification rate in cancer cells [23]. These data indicate that TWIST1 directly increases the expression of glycolytic genes, including GLUT1, suggesting its effectiveness as a potential therapeutic target [23]. In addition, one of the most important pathways during early stages of PDA development is PI3K/Akt, which is involved in progression and survival of tumor cells [131]. Thus, one therapeutic strategy could be to inhibit PI3K/Akt pathways by apigenin, a promising anticancer flavonoid with antiproliferative properties [24]. Treating PDA cells with apigenin significantly inhibits glucose uptake and decreases GLUT1 expression at both the mRNA and protein levels [25]. An apigenin-based supplementary diet in resectable colon cancer reduced the recurrence rate of neoplasia (NCT00609310, phase 2) [132]. Another promising target for PDA treatment is paraoxonase 2 (PON2), a regulator of PDA progression by stimulating glucose uptake via GLUT1 [26]. PON2 is also involved in the AMPK pathway [26]; thus, treatment with AMPK agonists, such as metformin, could achieve effective results in term of stopping cancer growth [26]. These results lay the foundation for further studies to evaluate the effective strength of GLUT1 targeting in a clinical setting.

## 4. Hexokinase 2 (HK2)

HKs are a family of enzymes involved in glucose metabolism by catalyzing the first step of glycolysis. The HK family includes four isoforms (HK1–HK4), but HK2 is the only one overexpressed in several cancers [133,134,135,136,137]. Glucose is phosphorylated by HK2 to obtain glucose-6-phosphate (G6P), the major precursor in glycolysis, glycogenesis, PPP and hexosamine biosynthesis. Since under physiological conditions it mostly functions at its maximal rate, even modest increments of HK2 levels have a significant impact on glucose metabolism as a whole [30,138]. Among glycolytic enzymes, HK2 prevailed in tumor cells rather than in stromal cells of primary and metastatic PDA [30].

Gene expression analysis has revealed an association between higher HK2 expression and tumor aggressiveness in PDA patients, suggesting the prognostic value of HK2 [30,31,32]; however, another study highlighted an inverse correlation [32]. The pivotal role of HK2 in primary tumor growth and metastasis in PDA was demonstrated by HK2 knockdown, which led to a decrease in tumor growth both in vitro and in vivo, while its overexpression correlated with reduced overall survival in patients [28]. Moreover, in cancer cells, the overexpression of HK2 directly regulated glycolytic activity, increasing lactate production which, in turn, promoted VEGF-A signaling, one of the pathways involved in invasion, cancer cell extravasation and colonization at distant organs [28].

HK2 also protects cancer cells from apoptosis [139]. In fact, hypoxic conditions promote transcription of hypoxia-inducible factor 1 alpha (HIF-1α), which binds c-Myc leading to the transactivation of HK2. Phosphorylated HK2 binds to the voltage-dependent anion channel 1 (VDAC1) located at the outer membrane of mitochondria. Thus, proapoptotic proteins cannot bind this channel, not allowing the escape of cytochrome c from mitochondria, suggesting that the interaction between HK2 and VDAC1 is required for counteracting cell apoptosis [140,141,142]. In PDA cells, GEM treatment increased HK2-VDAC1 binding through ROS production, and the consequent antiapoptotic effect could be related to GEM resistance; in fact, overexpression of HK2 in PDA patients correlated with GEM resistance [29].

A promising approach to target HK2 could be the use of ikarugamycin (IKA), a polycyclic tetramate macrolactam isolated from *Streptomyces xiamenensis 318*. Several studies have already reported the main biological activities of IKA, such as immune regulation [33] and cytotoxic [143,144] and antitumor activity [34]. On this basis, Jiang et al. [34] identified IKA as an HK2-selective small molecule inhibitor able to suppress the glycolytic phenotype of PDA cells, reducing glucose consumption and lactate production. The authors have also demonstrated the effectiveness of IKA in reducing tumor growth in vivo and in increasing GEM sensitivity in vitro. Taken together, these findings suggest IKA as a potential treatment for PDA patients used alone or in combination with first-line drugs. However, the contribution of the immune system, when HK2 is overexpressed, has not been fully elucidated. For example, in PDA no HK2 antibody response has been detected yet, but an increased level of anti-HK1 antibodies has been shown to be associated with unfavorable outcomes in primary biliary cholangitis patients [145,146].

## 5. Aldolase A (ALDOA)

The aldolase family members (ALDOA, ALDOB, ALDOC) are enzymes involved in glycolysis, catalyzing the conversion of fructose 1,6-bisphosphate into glyceraldehyde 3-phosphate (G3P) and dihydroxyacetone phosphate [147].

ALDOA is expressed in embryos and is abundantly present in adult muscle tissue, playing an important role in glycolysis and in maintaining glucose homeostasis [148,149]. ALDOA is not only expressed in normal tissue, but its overexpression has also been found in several human cancers, including oral squamous cell carcinoma, lung squamous cell carcinoma, renal cell carcinoma and colorectal cancer [150,151,152]. Regarding PDA, ALDOA is highly expressed in patients with poor prognoses, and pancreatectomy has shown few benefits and an increased metastatic potential [35]. In addition, through serological proteome analysis (SERPA), anti-ALDOA antibodies were detected in about 30% of PDA patients and, in a fraction of them, the GEM-based treatment further increased antibody titer [10].

Several studies have shown the ability of 18F-FDG PET/CT to measure the metabolic tumor burden in patients with different solid tumors [153,154,155,156]. In PDA patients, a correlation has also been demonstrated between the metabolic tumor burden and an abnormal genetic background [157]. In particular, patients with aberrant expression of CDKN2A/P16, TP53 and SMAD4/DPC4 exhibited higher levels of several metabolic enzymes, including ALDOA, compared to patients with only one of these mutations, suggesting a relationship between genetic mutations and aberrant metabolism in PDA [157].

Taking together, these data suggest that overexpression of ALDOA in PDA cells is responsible for increased glycolysis, high metastasis, a low survival rate and poor prognosis; thus, the possibility to target ALDOA in order to hinder the glycolysis pathway was investigated. ALDOA knockdown not only resulted in glycolysis inhibition, but also in HIF-1α suppression, suggesting a feed-forward loop in which HIF-1α induced expression of glycolytic enzymes and vice versa [36]. In vitro and in vivo experiments using the ALDOA inhibitor TDZD-8, 1,2,4-thiadiazole demonstrated a decreased cell proliferation and a reduction in tumor growth due to a lower glycolytic activity [36]. Another HIF-1α inhibitor used in vivo, TX-2098, led to a lower expression of the vascular endothelial growth factor (VEGF), which is involved in tumor growth and metastasis, and a reduced expression of glycolytic enzymes, including ALDOA, resulting in a prolonged survival on a xenograft model of PDA [37].

## 6. Triose Phosphate Isomerase (TPI) and Glyceraldehyde-3-Phosphate Dehydrogenase (GAPDH)

TPI catalyzes the interconversion of dihydroxyacetone phosphate (DHP) into G3P. TPI is highly expressed in several cancer types [158,159,160], including PDA [13], and plays a role in migration and invasion of tumor cells [9,13,38,161]. Meta-analysis has shown that TPI was increased in the proteomes and secretomes of PDA patients [38]. Proteomic analysis of PDA patient sera treated with GEM revealed that TPI serum levels were higher in patients with the worst prognoses, suggesting TPI as a putative prognostic marker of response to GEM treatment [13,39]. Of note, the presence of autoantibodies in response to TPI, which increased further after GEM treatment, was demonstrated in PDA patient sera, indicating the potential role of TPI autoantibodies as an immunological target [9,10].

In the glycolytic pathway, TPI is followed by GAPDH, the enzyme that catalyzes the phosphorylation of G3P into 1,3-bisphosphoglycerate. GAPDH has been identified on cell membranes and also in the nucleus, where it regulates the transcription of several genes, but its role remains elusive [43].

Protein analysis based on two-dimensional electrophoresis and liquid chromatography-mass spectrometry revealed that two isoforms of GAPDH, together with other glycolytic enzymes, were overexpressed in tumors compared to normal tissues from PDA patients [41]. In addition, GAPDH protein levels were elevated in human PDA cells compared to normal tissues [40], and PDA patients showed shorter disease-free survival times [39]. In a comparative analysis of human PDA and paired para-cancerous tissues, several peptides related to GAPDH were found to be differentially expressed. Among these peptides, P1DG significantly inhibited the proliferation and migration/invasion of PDA cell lines in vitro [44]. Furthermore, GAPDH seemed to be upregulated in metastatic lesions obtained from a PDA orthotopic mouse model [42]. Increased GAPDH mRNA and protein levels were found in nude mice after injection of human PDA cell lines when compared to normal pancreata [40].

This evidence, both in human tissue and in a mouse model, suggests that targeting GAPDH could be an effective strategy to counteract tumor progression. One interesting approach is based on the inhibition of the moderator of oxidative stress protein—uncoupling protein 2 (UCP2)—by genipin treatment, which enhances ROS production as well as the ROS-dependent antiproliferative and proapoptotic effects in PDA cells [45]. Genipin could induce nuclear GAPDH positivity in PDA cell lines inducing autophagy-related genes, as the formation of autophagic vesicles, while, in vivo, reductions in tumor masses were observed [45,46]. Furthermore, in the cytosolic compartment, GAPDH seemed to be related to mutated p53 (mutp53), which prevented nuclear translocation in PDA cell lines and promoted the cytosolic glycolytic activity of GAPDH [112]. Mutp53-dependent sirtuin-1 (SIRT1) expression caused the formation of complexes with GAPDH, which promoted its cytosolic stabilization. Moreover, the phosphorylation of GAPDH in different residues by the kinases AMPK and AKT resulted, respectively, in the inhibition or stimulation of GAPDH nuclear translocation [112]. Indeed, GAPDH nuclear localization could be restored by AMPK activation or AKT inhibition, and the mutp53-dependent inhibition of autophagy in PDA cell lines could be overturned [112]. The role of GAPDH in tumor progression has prompted several studies on the effects of GAPDH inhibitor molecules. Among them, it was found that AXP3009 could restore PDA cell proliferation and antiapoptosis signaling suppressed by genipin through tertiary alterations that affect GAPDH stability and abate its nuclear translocation, suggesting that in high levels of ROS, GAPDH could exert an antitumor effect on the nucleus [45]. Conversely, inhibiting GAPDH cytosolic activity in PDA cells by AXP3009 caused the reversal of mutp53-dependent proliferation and apoptosis inhibition [112]. This evidence identified—in the glycolytic pathway—a potential therapeutic approach for PDA patients who have an oncogenic mutation in p53. In fact, GAPDH silencing in mutp53 PDA cell lines restored GEM sensitivity [112]. PDA patients with wild type p53 could be treated with 2-Deoxy-D-Glucose (2-DG) by suppressing glycolytic activity to decrease GEM resistance-related genes and an increased apoptosis rate [39].

Focusing on the immune response in GEM-treated PDA patients, both humoral and cellular GAPDH-specific responses were observed [10]. Interestingly, GEM treatment further enhanced the specific antibody response [10] and the accumulation of GAPDH bound to IgM antibodies increased, correlating with the worst prognosis in PDA patients [48].

Koningic acid (KA), a specific GAPDH inhibitor, influenced the lactate production flux, thus glycolytic intermediates upstream of GAPDH were accumulated and fluxes in the downstream GAPDH pathway were dysregulated [47]. KA was tested in vitro on 60 cancer cell lines from different tissues subjected to heterogeneous cytotoxic effect [47]. Moreover, KA sharply reduced glycolytic flux in cells undergoing a higher degree of the Warburg effect than in cells with lower glycolysis, suggesting that KA activity is not due to individual genes, but to the global level of glucose metabolism [47]. Glucose uptake and lactate excretion significantly correlated with the KA response, suggesting the clinical applicability of glycolytic flux as a predictor of the KA effect. Moreover, in vivo experiments using orthotopically injected mice with KA-sensitive PDA cells showed that KA led to the suppression of tumor growth [47].

## 7. Forkhead Box Protein M1 (FOXM1)

FOXM1 is a member of the Forkhead box transcription factor superfamily, which consists of more than 50 members sharing a conserved winged-helix DNA-binding domain [162]. It is involved in cell proliferation, DNA damage repair, apoptosis, differentiation, and transformation [55,163,164,165,166]. Three different isoforms for human FOXM1, with different glycolytic activity, were isolated—namely, FOXM1 A, FOXM1B and FOXM1C [53]. FOXM1A is transcriptionally inactive, while FOXM1B and C isoforms are involved in PDA progression [53]. The isoform FOXM1B transcriptionally upregulated LDHA expression and increased its activity, lactate production and glucose utilization, and consequently promoted pancreatic tumorigenesis and metastasis [53]. Of particular interest, FOXM1C overexpression was shown to be involved in the epithelial-to-mesenchymal transition (EMT), migration, invasion and metastasis of PDA cells, whereas FOXM1 inhibition reverted these processes [56,58]. In addition, in PDA cells, elevated levels of FOXM1C upregulated uPAR, which plays a central role in angiogenesis and metastasis in different cancer types including PDA [56]. Activation of the uPA/uPAR axis in PDA tissues and cell lines correlated with an increase in FOXM1, and silencing of FOXM1 suppressed uPA and uPAR expression, angiogenesis and tumor growth both in vitro and in vivo [57].

Several studies have reported FOXM1 overexpression in human PDA cell lines and tumor specimens, as well as a correlation with clinical phenotype, lymph node metastasis, histological differentiation and poor survival [49,50,52], suggesting FOXM1 expression as a biomarker to predict the clinical outcome of PDA patients. FOXM1 in PDA was shown to be involved in the Warburg effect, modulating the expression of PGK1 and LDHA [53,56]. There is also evidence for a possible role of FOXM1 in self-renewal and proliferation of cancer stem cells (CSCs) [167,168] and overexpression of FOXM1 led to an increased self-renewal capacity of human PDA cells in vitro, which correlated with an enhanced CSC marker [162]. Indeed, FOXM1 plays a central role in the early stages of PDA development via crosstalk with signaling pathways related to PanIN (Ras/Raf/MAPK, PI3K/Akt) and PCSCs. Moreover, FOXM1 maintains the stem cell pluripotency in vivo by inducing the expression of Oct4, Nanog, and Sox2 [169]. A link between FOXM1 and HGF/Met signaling in PDA progression was also observed [170]. In fact, elevated levels of Met in different tumors correlated with increased expression of FOXM1, due to binding of FOXM1 to the promoter region of the Met gene [170] and the increased FOXM1 expression was shown to enhance the activation of HGF/Met signaling and its downstream pathways (RAS/ERK1,2, PI3K/Akt) [170].

Different therapeutic approaches have been used to counteract the increase in FOXM1 in cancers, including targeting of ubiquitin-specific peptidase 5 (USP5). USP5 is significantly overexpressed in a panel of PDA cell lines and positively associated with FOXM1 expression [59]. Inhibition of USP5 significantly decreased tumor growth and correlated with downregulation of FOXM1, suggesting that USP5 plays a critical role in tumorigenesis and progression of PDA by stabilizing the FOXM1 protein [59]. Thiazole antibiotics that specifically target FOXM1 inhibited cancer growth, inducing apoptosis [60]. The proteasome inhibitors, MG115 and MG132, were used to inhibit the transcription activity of FOXM1, favoring apoptosis. In vivo MG132 delayed the growth of BxPC3 cancer cells showing a reduction in tumor growth and angiogenesis by decreasing levels of NF-kB, VEGF and IL-8 [61,62]. 5,7 Dimethoxyflavone isolated from Kaempferia Parvoflora Wall, an anti-inflammatory molecule, inhibited the migration rate of pancreatic sphere-forming cells. This effect was due to changes in EMT biomarkers and to the modulation of both the FOXM1 and Sox2 genes [63]. A natural isoflavonoid found in soybean products, genistein, efficiently counteracted PDA cell growth and invasion in vitro reducing FOXM1 expression [55]. Three clinical trials (NCT00376948, phase 2–NCT01182246, phase ½–NCT02336087, phase 1) using genistein in combination with chemotherapy in PDA patients are ongoing and unfortunately, one trial (NCT00376948, phase 2) was stopped early due to the lack of efficacy.

FOXM1 also seemed to be involved in the resistance to paclitaxel therapy via the activation of the FOXM1/PHB1/RAF-MEK-ERK pathways and the enhancement of the ABCA2 transporter [51]. Indeed, targeting of FOXM1 led to an increase in sensitivity of PDA cells to paclitaxel [51]. Elevated FOXM1 levels were also found in patients resistant to GEM in which IFN-γ could downregulate FOXM1 by STAT1 phosphorylation [54].

Few studies have been conducted to better understand how the overexpression of FOXM1 can affect the compartment of immune cells. In particular, deletion of FOXM1 in dendritic cells (DCs) decreased cell surface expression of MHCII and CD86 in response to the house dust mite allergen, suggesting an important role of FOXM1 in DC maturation [64]. This is in accordance with high infiltration of DCs observed in tumors of patients with a poor survival [65]. Furthermore, FOXM1 altered maturation of bone marrow-derived dendritic cells, inhibited T cell proliferation and decreased IL-12 p70 in tumor-bearing mice [65]. Further experiments are necessary to dissect the relationship between the overexpression of FOXM1 and the TME in cancer progression.

## 8. Phosphoglycerate Kinase 1 (PGK1)

PGK1 generates a single ATP molecule in the glycolysis pathway catalyzing the conversion of 1,3-diphosphoglycerate to 3-phospoglycerate [171].

The enzyme PGK1 also participates in many biological activities, such as angiogenesis, autophagy and DNA repair, hindering the comprehension of its role during cancer development [67]. Interest in its mechanisms is intensified by the fact that PGK1 is one of the only two enzymes that is involved in ATP production during aerobic glycolysis in cancer cells [172].

PGK1 is overexpressed in various cancer types, including bladder, brain, breast, colorectal, head and neck, kidney, lung and ovarian cancers [67]. In addition, its overexpression is related to poor prognosis in cancer patients [67]. In PDA, PGK1 was not only more strongly expressed in > 70% of patients’ tumor tissues compared with nontumoral counterparts, but PGK1 levels in sera were also significantly higher in PDA patients compared to healthy controls, reflecting faster growth and more hypoxic tumors [66]. In fact, higher autoantibody levels against PGK1 were only detected in the sera of patients and were associated with an unfavorable prognosis [10,68,70].

PGK1 was identified as a direct target gene of the nuclear factor of activated T cells 5 (NFAT5), a transcription factor involved in many malignancies [173,174,175,176]. NFAT5 was overexpressed in PDA samples, both at the protein and mRNA levels, and it correlated with a poor prognosis in patients [173]. In fact, NFAT5 facilitates cancer cell survival, boosting a glycolytic phenotype and tumor progression via transcription of PGK1 [173].

During PDA development, PGK1 expression is inversely correlated with that of SMAD4, a specific tumor suppressor gene for PDA [69]. Since the loss of SMAD4 expression is responsible for increased glycolysis in cancer cells due to the overexpression of glucose transporters [177], the loss of SMAD4 in PDA cells also led to an increased expression of PGK1, enhancing glycolysis and more aggressive tumor progression [69]. Moreover, PGK1 localization could also be responsible for different behaviors: cytoplasmic PGK1 might act to promote growth of the primary tumor and instead nuclear PGK1 could enhance the spreading of metastasis [69].

All these data suggest the role of PGK1 as an oncogene in SMAD4-negative PDA patients and its presence could predict the metastatic fate of tumors, proposing this glycolytic enzyme as a potential therapeutic target.

## 9. Lactate Dehydrogenase A (LDHA)

LDHA converts pyruvate to lactate by oxidation of the reduced form of NADH to NAD^+^. LDHA mRNA and protein expression levels are elevated in PDA samples compared to the normal paired tissues [53,71,72,73,75]. In PDA patients, a positive association of LDHA expression with disease stage, tumor size [39,73,74,75] and differentiation was observed [73]. The LDHA staining score significantly correlated with the proliferation marker Ki-67 [71], and patients who expressed LDHA at higher levels displayed a worse survival [39,71,74]. Interestingly, a significant inverse correlation between the LDHA staining scores and CD8^+^ TIL count was observed in PDA patients [71]. By contrast, no antibody signature was observed in PDA patients, while high serum LDHA levels were detected in metastatic colorectal carcinoma patients, correlating with a shorter progression-free survival and overall survival time [178]. These data suggest that the analysis of LDHA levels in PDA patients’ sera could be an interesting biomarker to be investigated.

LDHA overexpression promoted proliferation in PDA cells [72,73,75] and its expression could be regulated by HIF-1α under hypoxia conditions [75,179,180,181]. Connections with adjacent cells could affect glucose consumption and tumor progression. Focal adhesion kinase (FAK) expression was significantly higher in PDA cell lines compared to normal cells and inhibition of FAK decreased LDHA levels [182]. Moreover, in a FAK-deficient xenograft model, a delay in tumor growth compared to wild type, with lower size and weight isolated tumors in FAK-deficient conditions was observed [182]. LDHA knockdown inhibited PDA cell growth [72,75], promoted cell migration [75] and downregulated the expression of antiapoptotic genes, such as XIAP and Bcl-2 [72], resulting in the attenuated tumorigenicity of PDA cells injected in mouse model [72].

There were significant positive correlations between mRNA levels of c-Myc and LDHA in both PDA cell lines and specimens [74]. Suppression of the c-Myc-LDHA axis inhibited tumor growth and progression in PDA cell lines, and the same effects were observed with 2-DG, a glycolysis inhibitor [74]. In addition to synthetic inhibitor molecules, epigallocatechin gallate (EGCG), the major biological active constituent of green tea, reduced lactate production and the glycolytic rate in PDA cells, suggesting an impact on the cancer metabolic network [76]. Of note, an ongoing clinical trial (NCT02336087, phase 1) aimed to treat PDA patients with tumors that cannot be removed by surgery by combining conventional chemotherapy treatment with EGCG as a dietary supplement in order to simultaneously block different targets in the cancer cell, which may slow down cancer growth. Furthermore, PDA cell lines treated with graviola extract displayed lower transcription levels of LDHA, and consequent decreased rates of glucose uptake and inhibition of ATP production were observed [77]. Graviola treatment resulted in a dose-dependent reduction in cell viability and in downregulation of the ERK, AKT and HIF-1α pathways [77]. Cell death induced by graviola seemed to be caused by necrosis, which was also observed in tumor-bearing mice [77]. Graviola could also affect PDA cell line motility and migration by disrupting the actin network and downmodulating phosphorylated FAK and MMP9 protein. In addition, MMP9 was also found to be reduced in in vivo experiments, in which graviola treatment led to partial PDA tumor eradication [77]. A recent clinical trial (NCT04773769), in the recruiting phase, studied the antitumoral effect of graviola in patients with gastric, pancreatic, colorectal, hepatocellular and lymphomas in which conventional chemotherapy had failed. Another potential therapeutical approach to inhibit LDHA is FX11, which caused a reduction in the proliferation of PDA cell lines and of tumor growth in vivo without side effects [78,79]. In greater detail, treatment of a PDA cell line with FX11 resulted in increased ROS production, cell death and, similarly, LDHA silencing increased oxygen consumption [79]. FX11 therapy was shown to induce a greater inhibition of tumor growth and apoptosis in a human TP53-mutant xenograft compared to wild type, probably due to the higher expression of TIGAR which favors the PPP instead of glycolysis [12]. These data highlight the TP53 status as a molecular key determinant of response to LDHA inhibition [12]. Finally, the LDHA-dependent lactate secretion could also inhibit antitumor immunity. Indeed, LDHA-deficient murine tumors displayed higher cytotoxic activity of NK cells compared to controls, and myeloid-derived suppressor cells (MDSCs) were decreased in tumors with lower suppressive effect, suggesting that LDHA activity could also impact the antitumor immune response through the higher paracrine signaling of tumor-derived lactate [80].

## 10. Alpha Enolase (ENO1)

ENO1 catalyzes the conversion of 2-phospho-*D*-glycerate into phosphoenolpyruvate in the ninth step of glycolysis. Elevated levels of ENO1 were found in many types of cancer, including PDA. ENO1 mRNA levels were higher in both human PDA cell lines and tumor tissue derived from the same cells injected into nude mice, compared to normal pancreata [40]. Microarray data generated from human PDA tissues and cell lines showed significant differential levels of ENO1 mRNA expression and protein levels in tumors compared to normal pancreata [81]. ENO1 tissue expression was higher in the nucleus and cytoplasm, but lower in the membrane [83]. Nevertheless, ENO1 was also detected on the cell surface of several PDA cell lines [82]. From two-dimensional electrophoresis (2-DE) protein analyses of human PDA tumor tissues, ENO1 resulted as being overexpressed compared to normal pancreatic tissues [41]; however, in PDA cell lines cultured under growth factor stress, ENO1 was downregulated compared to parental cells [183]. Interestingly, ENO1 was also found in the plasma of PDA patients at higher levels compared to normal subjects [83]. Patients with both high ENO1 plasma concentration and score in tumor tissue, had a positive association with tumor stage and poor prognosis [83,84]. TCGA data showed that patients with ENO1 overexpression had shorter disease-free survival times and ENO1 expression correlated with GEM tolerance-related genes [39]. Interestingly, PDA patient sera contained specific antibodies against metabolic enzymes and cytoskeletal proteins, which were upregulated in PDA tissues [9]. PDA patients treated with GEM showed enhanced ENO1-specific antibodies [10]. In particular, two out of six ENO isoforms (ENO1 and ENO2) were found to be preferentially expressed in PDA, and phosphorylated on serine 419, triggering a humoral response in more than half of PDA patients with a better clinical course and prognosis [90]. Moreover, ENO1 and ENO2 autoantibody levels were higher in patients with normal CA19.9 levels, thus complementing the efficiency of the serological test and achieving clinical relevance [90]. In addition, significant T cell proliferation, IFN-γ production and cytotoxic activity were found in healthy donors after in vitro coculture with ENO1-pulsed-DCs compared to controls [81]. Furthermore, PDA patients with a higher percentage of circulating ENO1-specific T cells and anti-ENO1 antibodies showed a longer survival [91]. A correlation between ENO1-specific T cells and the anti-ENO1 IgG response was observed in PDA patients [81]. ENO1-specific T helper 17 clones isolated from PDA patients’ tumors or normal pancreata promoted an antitumor-specific immune response, and were decreased in tumors compared to healthy mucosa, while elevated levels of ENO1-specific Treg clones were observed in the tumoral area [92]. Analysis of the ENO1-specific peripheral and tumoral T cell receptor repertoire demonstrated that, in PDA patients, T cells could recirculate from the tumor to the periphery [91].

ENO1 expression could be regulated by different molecular mechanisms. FAK deficiency resulted in lower ENO1 levels, delaying the in vivo tumor growth [182]. Moreover, the transcription factor, TWIST1, is a crucial regulator of Warburg metabolism of PDA cells, and it was demonstrated that TWIST1 transcriptionally regulated ENO1 [23]. ENO1 silencing led to impaired glycolytic flux and decreased levels of lactate, but increased glucose uptake [88]. In hyperglycemic conditions, glucose could force alternative metabolic pathways to induce oxidative stress [88].

Since ENO1 is involved in the plasminogen-dependent invasion of PDA cells, which express uPAR—a plasminogen activator—on their cell surfaces, an effective strategy used in PDA preclinical model is based on ENO1 silencing and anti-ENO1 mAb [82]. Data showed a significant reduction in plasminogen-dependent invasiveness in vitro [82] and a smaller metastatic area, together with a decrease in tumor growth in vivo [82,85,88]. The same effect was observed in tumor-bearing mice treated with anti-ENO1 mAb or injected with adeno-associated virus expressing anti-ENO1 mAb [82]. This decrease in metastasis could be due to the dysregulated isoform of integrins interacting with uPAR to activate an intracellular signaling pathway involved in senescence and ROS production [85]. In additional studies, anti-ENO1 mAb, which binds ENO1 expressed on the surface of MDSCs, inhibited adhesion to endothelial cells and migration in vitro and in vivo, while enhancing the secretion of proinflammatory cytokines, such as IL-6 [87]. Moreover, anti-ENO1 mAb increased IFN-γ and IL-17 while decreasing IL-10 and TGF-β production by activated T cells, suggesting its potential role in limiting MDSC infiltration into the TME [87]. In addition, in PDA cell lines ENO1 silencing impaired cell proliferation, enhanced apoptosis, modulated the actin reorganization from the membrane towards the cytoplasm and increased intracellular ROS, thus targeting ENO1 to modulate the redox homeostasis restored chemosensitivity to GEM [84,85]. The pharmacological inhibition of ENO1 with phosphonoacetohydroxamate (PhAH) was selective to inhibit enzymatic activity in cancer cells, where PhAH also significantly reduced proliferation; instead, in normal human fibroblasts or normal pancreatic cells, PhAH was not effective [88]. Immunization with citrullinated ENO1 peptides in mice challenged with PDA cells induced Th1 responses and a longer survival compared to the control group [89]. Another therapeutic treatment tested in a preclinical model was the ENO1 DNA vaccination, which increased survival rate [10,86], reducing the presence of MDSCs and Treg cells, while increasing Th1-, Th17- and ENO1-specific antibody responses, thus mediating antitumor activity [86]. In addition, GEM treatment unleashed CD4 antitumor activity and strongly impaired tumor progression compared with mice that were vaccinated or GEM-treated [10]. Surprisingly, antibody and T cell responses to GAPDH were induced by ENO1 vaccination and further increased by GEM, suggesting an epitope spreading effect [10]. Taking these together, promising results have been obtained in preclinical models, suggesting that ENO1 could be a potential target, for validation in clinical trials.

## 11. Pyruvate Kinase M2 (PKM2)

PKM2 has an important role in the Warburg effect and in cancer cell metabolic reprogramming. Pyruvate kinase catalyzes the formation of pyruvate and ATP from phosphoenolpyruvic acid (PEP) and ADP. A large number of studies have reported that the overexpression of PKM2 correlates with a poor prognosis in different cancer types including PDA [31,39,71,95,100,184,185], while other reports have shown the inconsistency of this correlation [95,96,186]. Possible explanations for these contrasting results are differences in enrollment for the patients, sample size and programs used to analyze data. Interestingly, an increased autoantibody response to PKM2 was observed in about 60% of PDA patients, and GEM treatment further enhanced the humoral response proportionally to chemotherapy cycles [10].

To better investigate the effective role of PKM2 in PDA progression, different approaches have been used. Human or murine PDA cell lines were silenced or knocked down for the PMK2 gene, demonstrating its involvement in metastasis, proliferation, migration and tumor formation [93,99,100,101,186]. From a molecular point of view, serine/threonine protein kinase 2 (PAK2) has emerged as a possible target of PKM2; indeed, PKM2 knockdown decreased PAK2 protein half-life by increasing ubiquitin-dependent proteasomal degradation [99]. PKM2 and PAK2 protein expression positively correlated with each other in PDA tissues and metastasis, suggesting this complex as a possible target [99]. Moreover, deletion of PKM2 impaired proliferation, increased apoptosis, and decreased PDA tumor growth by negatively impacting both HIF-1α and VEGF secretion through the NF-kB/p65, mTOR and Akt/c-Myc pathways [46,93,179,187]. The PKM2 gene was involved in MAPK signaling [188,189], and its critical role related to STAT3 and TWIST1 was suggested, with particular reference to the EMT, in increasing invasion and metastasis [23,100]. In fact, silencing of the PKM2 gene induced apoptosis, impaired autophagy and decreased PDA tumor growth in vivo [94,97]. FAK was found aberrantly overexpressed or activated in PDA, increasing the expression of key glycolytic proteins, including PKM2 and the monocarboxylate transporter [182]. Furthermore, active/tyrosine-phosphorylated FAK directly bound to PKM2 and promoted PKM2-mediated glycolysis [182].

Different inhibitors were used to block or limit the activity of PKM2, such as treatment with a glycolysis inhibitor, 2-DG or (*R,R’*)-4′-methoxy-1-naphthylfenoterol, in both cases reductions in tumor proliferation, cancer cell survival and invasion were observed [102,190]. Shikonin is another PKM2 inhibitor and it was able to reduce PDA cell proliferation, cell migration and to induce cell death through the inhibition of glycolysis, ATP depletion, inhibition of plasma membrane calcium pump (PMCA) and cytotoxic Ca^2+^ overload [103]. PDA cells shifted their metabolism towards glycolysis, which fuels the PMCA, thereby preventing Ca^2+^-dependent cell death [103]. A clinical trial on bladder urothelial carcinoma patients (NCT01968928) is ongoing to evaluate the use of shikonin in combination with chemotherapeutic agents to regulate cytotoxic effect of chemotherapy and overcome chemoresistance. Fatty acid synthase (FASN) overexpression caused resistance to GEM-based therapy in PDA, and it directly regulated PKM2 expression and glucose metabolism, leading to GEM chemoresistance in PDA cells [191]. Dietary compounds such as betulinic acid and thymoquinone, in combination with GEM, downregulated the expression of PKM2, inhibited cell proliferation by inducing apoptosis and potentiated cytotoxicity [192]. Moreover, knocking down of PKM2 also significantly enhanced GEM-induced apoptosis by activating caspase 3/7 and PARP cleavage [104]. PKM1, the splicing variant of PKM2 derived from the same pyruvate kinase gene, increased the number of apoptotic cells in a colorectal cancer cell line [193,194]. Alternative splicing of the pyruvate kinase gene was differentially modulated in GEM-resistant PDA cells, resulting in the promotion of the PKM2 isoform, whose expression correlated with short recurrence and disease-free survival in patients [98]. The switch toward the PKM1 variant rescued sensitivity of GEM-resistant PDA cells, suggesting that PKM2 expression is required to overcome GEM-induced genotoxic stress [98].

## 12. Metabolic Reprogramming to Increase the Immune Response in Tumors

**Figure 2:**
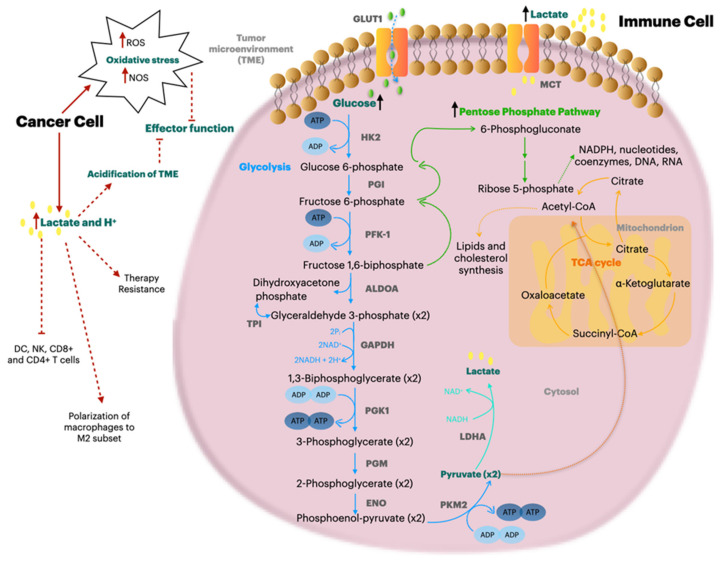
Metabolism of activated T cell and the effect of cancer-induced tumor microenvironment (TME) acidification on T cell response. High glycolytic cancer cells strongly consume glucose available in the TME and in return they produce massive quantities of lactate. TME acidification and lack of nutrients, together with the higher cancer-dependent reactive oxygen species (ROS) production, impair the activation of T effector cells. T cell activation requires higher level of glucose to support an increased glycolytic rate, and the derived metabolites are also needed for pentose phosphate pathway (PPP) and lipidic synthesis. Red arrows indicate the effects of altered cancer metabolism on T cell activity and black arrows indicate the involved pathway in T cell activation.

Several reports have shown that targeting the glycolytic pathway can effectively counteract tumor progression, which could also be due to the role of cells that are part of the TME. Increasing needs of nutrients and glucose in tumors could shift immune cells (Figure 2) and stromal cells towards glycolytic behavior [195]. For example, PDA cancer-associated fibroblasts with a glycolytic phenotype supported oxidative phosphorylation in cancer cells and, consequently, tumor survival and invasive ability [196]. The same phenomenon was observed in PDA pancreatic stellate cells via HK2 expression [195,197]. One of the mechanisms of the metabolic interaction of stromal and tumoral cells is through the lactate efflux, thus lactate transporters may play a pivotal role [198]. In greater detail, the lactate transporter MCT-4 was associated with invasiveness and migration of PDA cells [199]. Of note, in PDA, the effect of inhibition of glycolytic enzymes on the immune compartment has not yet been fully investigated.

The recruitment of macrophages has been previously observed in KRAS-mutated PDA cells by chemokine secretion [11]; in addition, CCL18 secreted by protumoral M2 macrophages induced aerobic glycolysis in PDA cells, promoting tumor survival and, in turn, the increase in lactate facilitated the conversion of M0 macrophages to M2 macrophages [196,200]. In addition, cytotoxic NK activity was inhibited by PDA cell-secreted lactate in an LDHA-dependent way [80,201,202].

Glucose metabolism also plays a pivotal role in suppressive functions and stability of Tregs in the TME. In fact, HIF-1α avoided glucose-derived pyruvate from mitochondrial activation promoting glycolysis, which was associated with a reduced suppressive function [203]. Glycolytic activity modulated FOXP3 expression which regulated Tregs stability [27]. In a glycolytic environment, ENO1 was directed towards the cytoplasm, avoiding binding with FOXP3 in the nucleus, thus preventing the Tregs suppressive phenotype [27]. In addition, silencing ENO1 in induced Tregs (iTregs) led to the reduction in FOXP3 expression as a consequence of lower ENO1 glycolytic activity, demonstrating that suppression of iTregs was mediated by the glycolytic enzyme ENO1 [203]. In the PDA glycolytic context, overexpression of GLUT1 and ENO1 was observed in Tregs, and their activation was due to glucose uptake [27,92].

Regarding the effector T cell subset, aerobic glycolysis is required for clonal expansion, cytolytic activity and cytokine secretion [204]. In CD8^+^ T cells, the altered metabolism impaired their effector function, promoting tumor progression [205]. Impaired glycolysis of CD8^+^ TIL was previously shown in cancers, leading to a lower proliferative rate and higher ROS production [206]. In a melanoma preclinical model, CD8^+^ TILs were found to be metabolically compromised due to a ENO1 post-transcriptional regulation since it was expressed as mRNA and protein level, but ENO1 enzymatic activity was defective [207]. In addition, in PDA patients, CD8^+^ TILs were negatively correlated with LDHA and PKM2 expression [71], while T cell involvement was reported for GLUT1 [18], FOXM1 [65] and ENO1 [81,91]. Moreover, tumoral T cell metabolism was affected by immune checkpoint signaling; indeed, PD1 expression inhibited the glycolysis pathway shifting the metabolism towards lipolysis and fatty acid oxidation [208]. Of note, the use of the immune checkpoint blockade was able to inhibit glycolysis in tumor cells, together with the enhancement of CD8^+^ TIL glycolysis and effector function, such as IFN-γ production [205]. In addition, anti-PDL1 synergized with the ENO1 inhibitor for enhancing autologous tumor-specific CD8^+^ and NK cell activity [209].

The relevance of targeting glycolytic enzymes has been demonstrated with the ENO1 vaccination in PDA preclinical mouse models. Indeed, this approach was shown to lead to a reduction in tumor burden by inducing both antibody and cellular responses [86]. Of note, activated MDSC expressed ENO1 on the surface at higher levels, and T cells in the presence of anti-ENO1-mAb-treated MDSC increased IFN-γ and IL-17 secretion, while IL-10 and TGF-β were decreased [87]. Moreover, chemotherapy treatment potentiated ENO1 vaccination efficacy [10].

## 13. Conclusions

Glycolytic proficiency may negatively affect survival outcomes of PDA patients. Altering cancer metabolism can significantly influence carcinogenesis, and the increased knowledge of these processes highlights them as potential therapeutic targets. Drugs that inhibit glycolytic enzymes or linked pathways have revealed encouraging effects in PDA therapy. Nevertheless, other studies are needed to clarify the involvement of the immune system in glycolytic enzyme overexpression. Metabolic manipulation not only acts on tumor progression, but could also enhance the immune cell fitness, thus improving immunotherapy approaches. The effectiveness of glycolytic enzyme targeting in combination with chemotherapy is under investigation in ongoing clinical trials in cancer patients whose survival outcomes are not yet known. However, preclinical studies show that GEM-based chemotherapy increased antibodies recognizing tumor-associated antigens (TAAs), including glycolytic ones, in a subset of PDA patients, rendering them potential therapeutic targets. In addition, data from PDA patients treated with GEM show an increase in the coordinated immune response to TAA, and this may offer new therapeutic and personalized options by targeting one or more TAAs that are recognized by each individual patient.

## Figures and Tables

**Table 1:** Schematic summary of potential glycolytic targets in Pancreatic ductal adenocarcinoma (PDA) therapy.

Target	Overexpression		Clinical Outcome			Targeting Approach	Correlation with Immune Response
	Cell Line	Tissue	Survival	PD/M	CT Resistance		
**KRAS/TP53**		PCR [11] IHC [11,12] RNAseq [13]	↓ [11,14]	↑ M [11]		KRAS interference [11] KRAS/p53 peptide vaccination [11,15,16,17,18] RT11-i [19]	Chemokine secretion, macrophage recruitment and lymphocyte and myeloid cell infiltration [11]
**GLUT1**	qPCR [20] WB [20,21]	RNAseq [15,16] IHC [17,18]	↓ [22]	* ↑ M [21]	[22]	GLUT1 silencing [21] TWIST1 silencing [23] Apigenin [24,25] PON2 [26]	Positive correlation with PD1^+^ TILs [18] High GLUT1 expression in activated Tregs [27]
**HK2**	qPCR [28] WB [28,29]	mRNA [30] IHC [30,31,32] Microarray [33]	↓ [29,31] ↑ [32]	* ↑ M [28]	[28]	HK2 silencing [28] IKA [34]	
**ALDOA**		IHC [35]	↓ [35]	* ↑ M [35]		ALDOA silencing [35] TDZD-8 [36] TX-2098 [37]	Circulating auto-Ab to ALDOA [10]
**TPI**	RNAseq [13]	RNAseq [13] IHC [38]	↓ [13,38]		[13,39]		Circulating auto-Ab to TPI [9,10]
**GAPDH**	mRNA [40] WB [40]	2-DE [41] IHC [41] mRNA [40] WB [40]	↓ [39]	* ↑ M [42]	[39]	2-DG [43] P1DG [44] AXP3009 [45] Genipin [45,46] KA [47]	Humoral and cellular responses to GAPDH [10,48]
**FOXM1**		Microarray [49] mRNA [50] IHC [51] WB [51]	↓ [50,52]	↑ PD [53] ↑ M [50,53]	[51] * [54]	Genistein [55] FOXM1 siRNA [56,57] FOXM1 RNA interference [58] USP5 inhibition [59] Thiazole antibiotic [60] MG115 and MG132 [61,62] 5,7 Dimethoxyflavone [63]	DC maturation [64,65] T cell proliferation [65]
**PGK1**	2-DE [66]	mRNA [67] 2-DE [66]	↓ [67,68]	↑ M [69]	[67]		Circulating auto-Ab [10,66,68] High concentration of PGK1 in serum [70]
**LDHA**	WB [71,72,73,74] qPCR [72]	Microarray [71] IHC [71,74,75] mRNA [72] WB [72]	↓ [71]	↑ PD [75]		LDHA RNA interference [72] LDHA siRNA [73,74] EGCG [76] 2-DG [76] Graviola [77] FX11 [78,79]	Negative correlation with CD8^+^ TIL [71] * Low cytotoxic NK activity [80] * High MDSC activity [80]
**ENO1**	mRNA [40] WB [40] Microarray [81] Flow cytometry [82]	2-DE [41] mRNA [40] WB [40] Microarray [81] IHC [83]	↓ [39,83,84]	* ↑ M [85]	[39,84]	ENO1 DNA vaccination alone or in combination with GEM [10,86] ENO1 silencing [82,85] Anti-ENO1 mAb [82,87] PhAH [88] ENO1 knockdown [88] Citrullinated ENO1 peptides [89]	Increased level of anti-ENO1 Ab before and after GEM treatment [9,10,90,91] Increased T cell response in vitro and in vivo [81,91] Increased level of ENO1 in plasma patients [83] ENO1-specific Th17 and Treg response [92]
**PKM2**	WB [71,93,94] qPCR [94]	IHC [31,71,93,94] ↓ IHC [95,96] Microarray [97] Oncomine database [97]	↓ [31,71,97,98]	↑ M [31,99] * ↑ PD [98]	[98]	PKM2 knockdown [94,99,100,101] PKM2 silencing [97] 2-DG [102,103] Shikonin [103] Betulinic acid and thymoquinone and GEM [104]	Increased level of anti-PKM2 Ab before and after GEM treatment [10] Negative correlation with CD8^+^ TIL [71]

CT—chemotherapy; PD—progressive disease; M—metastasis; *—preclinical mouse model; Ab—antibody; IHC—immunohistochemistry; WB—Western blot; qPCR—quantitative PCR; 2-DE—2-dimensional electrophoresis.

## Data Availability

No new data were created or analyzed in this study. Data sharing is not applicable to this article.
